# Evaluation of textural-based radiomics features for differentiation of COVID-19 pneumonia from non-COVID pneumonia

**DOI:** 10.1186/s43055-021-00592-0

**Published:** 2021-09-03

**Authors:** Yunus Soleymani, Amir Reza Jahanshahi, Maryam Hefzi, Mona Fazel Ghaziani, Amin Pourfarshid, Davood Khezerloo

**Affiliations:** 1 Medical Radiation Sciences Research Group, Tabriz University of Medical Sciences, Tabriz, Iran; 2 Department of Radiology, Faculty of Allied Medical Sciences, Tabriz University of Medical Sciences, 5166615739 Tabriz, Iran; 3 Department of Radiology, Faculty of Medicine, Imam Reza Hospital, Tabriz University of Medical Sciences, Tabriz, Iran; 4 Department of Radiology, Sina Hospital, Tabriz University of Medical Sciences, Tabriz, Iran; 5 Department of Medical Physics, Faculty of Medicine, Urmia University of Medical Sciences, Urmia, Iran

**Keywords:** COVID-19, Radiomics, Pneumonia, Chest CT

## Abstract

**Background:**

The false-positive rate of computed tomography (CT) images in the diagnosis of coronavirus disease 2019 (COVID-19) is a challenge for the management in the pandemic. The main purpose of this study is to investigate the textural radiomics features on chest CT images of COVID-19 pneumonia patients and compare them with those of non-COVID pneumonia. This is a retrospective study. Some textural radiomics features were extracted from the CT images of 66 patients with COVID-19 pneumonia and 40 with non-COVID pneumonia. For radiomics analysis, the regions of interest (ROIs) were manually identified inside the pulmonary ground-glass opacities. For each ROI, 12 textural features were obtained and, then, statistical analysis was performed to assess the differences in these features between the two study groups.

**Results:**

8 of the 12 texture features demonstrated a significant difference (*P* < 0.05) in two groups, with COVID-19 pneumonia lesions tending to be more heterogeneous in comparison with the non-COVID cases. Among the 8 significant features, only two (homogeneity and energy) were found to be higher in non-COVID cases.

**Conclusions:**

Textural radiomics features can be used for differentiating COVID-19 pneumonia from non-COVID pneumonia, as a non-invasive method, and help with better prognosis and diagnosis of COVID-19 patients.

## Background

In December 2019, a new human virus species, called coronavirus 2019, was identified in Wuhan, Hubei Province, China which causes a disease named “COVID-19”. The virus can rapidly spread and cause acute respiratory syndrome due to the presence of pneumonia. On March 11, 2020, the WHO declared the disease as a pandemic due to its high prevalence. Clinical signs of COVID-19 are fever (85%), cough (70%), and shortness of breath (43%) as well as gastrointestinal and abdominal symptoms. In some cases, it can even be asymptomatic. The overall mortality rate for COVID-19 is 2.3% [1, 2]. Real time-polymerase chain reaction (RT-PCR) is the standard method for diagnosing the COVID-19 with high specificity, but it has some limitations. This test has a relatively high rate of false-negative due to mistakes in sampling and low sample size; therefore, its sensitivity is low (about 59–71%). Moreover, PCR is a time-consuming test and the limitation in the number of PCR kits delay the testing process, which can lead to delayed diagnosis and early initiation of quarantine during the pandemic [2, 3]. Computed tomography (CT) scan with high sensitivity is another method for early detection of COVID-19 and can compensate the low sensitivity of PCR. CT scan sensitivity is high (about 88–98%). Moreover, CT is a feasible modality with a short scan time and long throughput time [3–6]. By combining CT with PCR, the COVID-19 can be diagnosed earlier.

After the prevalence of COVID-19, extensive studies of lung CT images have been conducted to differentiate COVID-19-induced pneumonia from other causes of pneumonia, which are based on the visual evaluation of images [2–13]. The CT scan for diagnosing COVID-19 can show unusual pneumonia, often peripheral, with the bilateral distribution in the lungs. The patchy ground-glass opacities (GGOs) and consolidations are the most common findings of chest CT scan for the COVID-19. The GGO pattern is often multifocal, bilateral, peripherally extended, and consolidated due to interlobular and broncho vascular septal thickening [11, 12]. In the early stages of the disease, GGO may appear as a single focal lesion, mostly in the lower lobe of the right lung [10, 14].

The most common morphological patterns of GGOs are patchy and rounded ones followed by triangular and linear ones. Some studies have been reported that an angular GGO with pleural thickening is a specific radiological sign of the COVID-19. Crazy paving is also common due to thickened interlobular septa with intralobular reticulations [3–5]. The prevalence of GGO and consolidation on the chest CT scan in COVID-19 patients are 88% and 32%, respectively. Moreover, bilateral lung involvement and peripheral opacities are observed in 87% and 76% of the cases [4].

The findings of CT scan can be changed as the disease progresses. In the early stages of COVID-19 infection, small patchy GGO with thick vascular lumens is more common, although, 56% of patients have normal CT images in the first two days after the onset of clinical symptoms (fever, dry cough, etc.). In cases of disease progression, multiple GGOs, and some severe cases, the patients may have diffuse lesions in both lungs that appear as a "white lung". When the patient recovers, the pulmonary GGOs disappear in the CT image, and the squamous pattern of GGO and parenchymal fibrosis are not observed [4, 10, 14].

The major limitation of CT scan in diagnosing COVID-19 and differentiating it from other types of pneumonia is its low specificity (about 34%) [4, 6]. So, it seems to be very important to find new methods in medical imaging such as texture analysis, mathematical techniques to extract significant features from an image at different gray levels, assessing the heterogeneity of lesions, and improving the early diagnosis [15].

Radiomics, as a completely non-invasive method for quantitative analysis of medical images, has recently received considerable attention. The radiomics method objectively characterizes multiple types of lesions using the advanced quantitative features called “radiomics features” of medical images. These features are divided into two general categories: semantic and agnostic features. Semantic features are used to describe morphologic characteristics of lesions such as shape, size, location, etc., while agnostic features (e.g. textural features) use innovative mathematical procedures in a high-throughput way that may fail to be perceived by the naked eye [16–19].

This study aimed to explore the association of textural radiomics features with COVID-19 pneumonia and non-COVID pneumonia on lung CT scans; that may provide a noninvasive means to profound recognition of the radiomics pattern of COVID-19 pneumonia as well as help to differentiate between COVID and non-COVID patients.

## Methods

### Study participants

This is a retrospective study conducted on 106 patients referred to our hospital from Jun-August 2020. After obtaining ethical approval from the institutional review board and patient informed consent for participation, participants were divided into two groups of patients with COVID-19 pneumonia (*n* = 66) and non-COVID pneumonia (*n* = 40). The inclusion criteria for COVID-19 patients were: Diagnosis of COVID-19 using RT-PCR, non-contrast CT at the time of diagnosis, and COVID-19 viral pneumonia at chest CT. The exclusion criteria were: Contrast CT exam and exams without slice thickness of 5 mm. For non-COVID patients, the inclusion criteria were: Patient with influenza virus pneumonia with signs and symptoms similar to the COVID-19 cases and who had non-contrast CT. Non-viral pneumonia, contrast CT exam, and exams without slice thickness of 5 mm were considered as exclusion criteria for non-COVID patients. Table 1 shows demographical information of patients enrolled in this study.

**Table 1:** The characteristics of patients in this study

Characteristics	Covid-19 pneumonia	Non-covid pneumonia
Number of patients	66	40
Age (mean ± SD)	50 ± 3.75	45 ± 2
*Gender*		
Male	30	18
Female	33	22

### Imaging protocol

Non-contrast CT was performed on all patients using a 16-slice single-source CT scanner (Somatom Sensation 16, Siemens, Forchheim, Germany). The tube voltage, tube current, and tube rotation time were 120 kVp, 150 mA, and 0.8 s, respectively. The slice thickness of each CT scan was 5 mm. The pitch was set at 1.5 and there was maximum inspiration breath-hold acquisition. The reconstruction matrix size was 512 × 512 pixels.

### Lesion segmentation

**Fig. 1:**
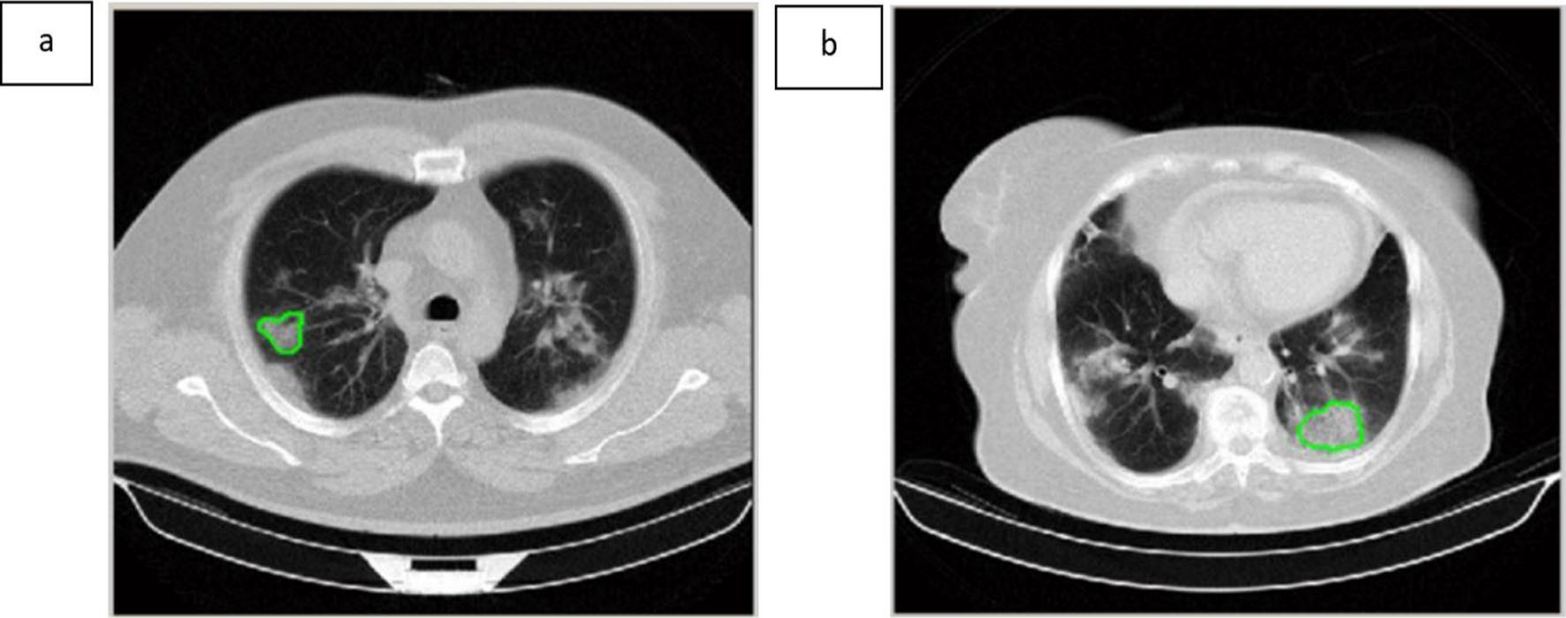
Non-contrast CT scan of (**a**) A 50-year-old man with COVID-19: the values of the cluster tendency, contrast, correlation, dissimilarity, energy, entropy, homogeneity, and gray-level nonuniformity were 134.237, 55.598, 0.413, 5.696, 0.003, 8.348, 0.262, and 0.037, respectively; **b** An 81-year-old healthy woman: the values of the cluster tendency, contrast, correlation, dissimilarity, energy, entropy, homogeneity, and gray-level nonuniformity were 40.201, 22.608, 0.281, 3.743, 0.006, 7.708, 0.324, and 0.027, respectively

To reduce the time of radiomics analysis, the region of interest (ROI) was manually localized inside a GGO on only one CT image slice by a radiologist with 10 years of experience in lung CT imaging who was unaware of the clinical results. One axial image among the chest CT images of each patient was selected and an ROI was drawn along the margin of the lesion (Fig. 1). In cases with multiple lesions, the lesion with a bigger size and/or higher density was chosen for ROI delineation.

### Radiomics features

Radiomics features provide quantitative information about medical images to assess various types of disorders. Textural features in this study were extracted from each ROI, as defined by Haralick et al. [20]. The texture is characterized by the spatial distribution of intensity levels in a neighborhood and evaluates the repeating pattern of local variations in image intensity. Eight gray-level co-occurrence matrices (GLCMs) including cluster shade, cluster tendency, contrast, correlation, dissimilarity, energy, entropy, and homogeneity were obtained for each lesion. A GLCM size of *N*_*g*_ × *N*_*g*_ defines the second-order joint possibility function of an image region constrained by the mask and is defined as $P\left( {{\fancyscript{i}},j|\delta ,\theta } \right)$. The (*i*, *j*)th element of this matrix characterizes the number of times the combination of levels *i* and *j* occurs in two pixels in the image separated by a distance of δ pixels along angle θ. The distance δ from the center voxel is defined as the distance based on the infinity norm.

Four gray-level run-length matrices (GLRLM) including gray-level nonuniformity, long-run emphasis, run-length nonuniformity, and short-run emphasis were also obtained for each lesion. A GLRLM is defined as the length in the number of pixels, of successive pixels that have the same gray level value. In a GLRLM $P\left( {{\fancyscript{i}},j|\delta ,\theta } \right)$, the (*i*, *j*)th element defines the number of runs with the gray level *i* and the length *j*, occurring in the image ROI along angle θ. All textural features were extracted from 128 bins in the intensity range of the whole image. The ROI extraction and radiomics feature calculations were performed in the S-IBEX, a free open-source radiomics software (https://github.com/abettinelli/SIBEX_Source) [21].

### Statistical analysis

To compare the differences in textural features of patients with and without COVID-19 pneumonia, the Mann–Whitney *U* test was performed in SPSS v.26 software. *P* < 0.05 was considered as the statistically significant level.

## Results

**Fig. 2:**
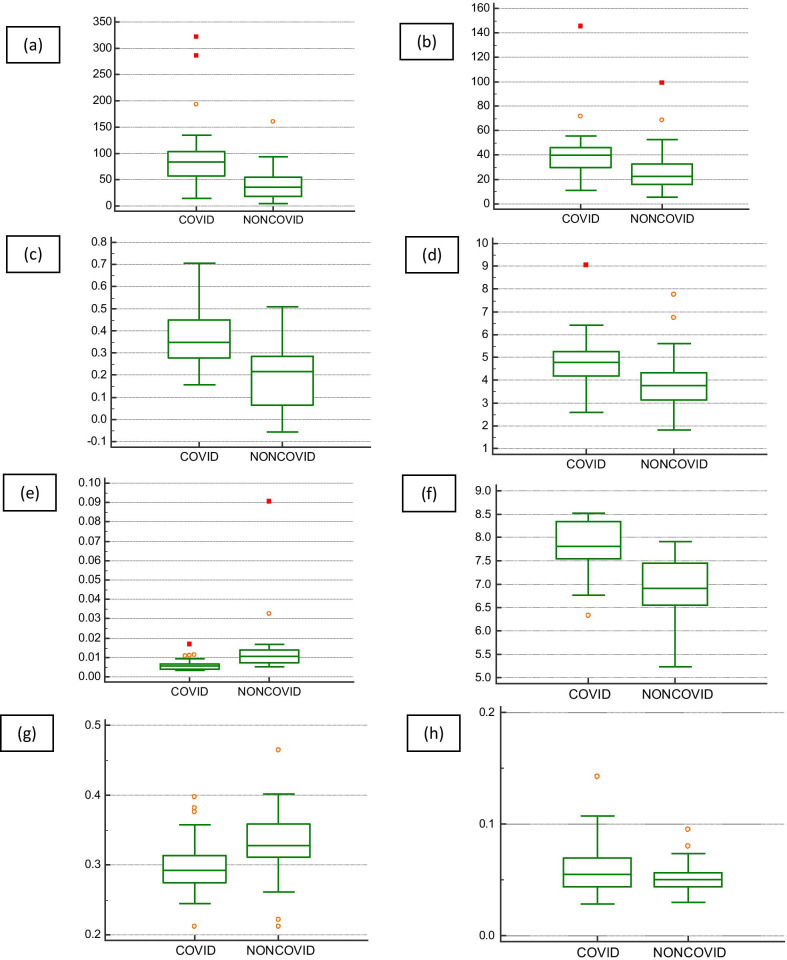
Box and whisker plots for the associations between COVID-19 status and the CT scan-based radiomics features. (**a**) cluster tendency, (**b**) contrast, (**c**) correlation, (**d**) dissimilarity, **e** energy, **f** entropy, **g** homogeneity and **h** gray-level nonuniformity

The average values and SD of the textural radiomics features are compared in Table 2. No statistically significant differences were observed in cluster shade of GLCM and long-run emphasis, run-length nonuniformity, and short-run emphasis of GLRLM between two groups (*P* > 0.05). Figure 2 shows the box and whisker plots of the radiomics feature values of two groups for *P* < 0.05.

**Table 2:** Feature parameters in association with COVID19 status

Feature parameters	Mean ± SD		*P* value
	COVID19 pneumonia	Non-COVID19 pneumonia	
*GLCM*			
Cluster shade	− 84.837 ± 824.714	− 152.944 ± 217.402	0.545
Cluster tendency	92.763 ± 64.802	45.132 ± 36.703	< 0.001
Contrast	40.825 ± 22.964	29.391 ± 22.267	0.007
Correlation	0.365 ± 0.140	0.180 ± 0.154	< 0.001
Dissimilarity	4.732 ± 1.164	3.982 ± 1.407	0.006
Energy	0.006 ± 0.002	0.015 ± 0.018	< 0.001
Entropy	7.807 ± 0.564	6.922 ± 0.721	< 0.001
Homogeneity	0.299 ± 0.039	0.329 ± 0.057	0.009
*GLRLM*			
Graylevelnonuniformity	0.060 ± 0.024	0.052 ± 0.024	< 0.001
Long run emphasis	1.404 ± 0.117	1.392 ± 0.128	0.956
Run-length nonuniformity	0.717 ± 0.051	0.728 ± 0.066	0.811
Short-run emphasis	0.916 ± 0.018	0.920 ± 0.022	0.811

For the GLCM features, cluster tendency, contrast, correlation, dissimilarity, energy, entropy, and homogeneity showed statistical significance between the two groups (*P* = <0.001, 0.007, < 0.001, 0.006, < 0.001, < 0.001, 0.009).

The value of cluster tendency, contrast, correlation, dissimilarity, and entropy in the COVID-19 pneumonia group was significantly larger than those in the non-COVID pneumonia group (Table 2; Fig. 2a–d, and f). On the contrary, the energy and homogeneity of the non-COVID pneumonia cases were larger than that of COVID-19 pneumonia (Table 2; Fig. 2e, g).

In the four GLRLM parameters, only one feature, gray-level nonuniformity, indicated statistical significance in differentiating the COVID-19 pneumonia from the non-COVID pneumonia patients (*P* < 0.001). As shown in Table 2 and Fig. 2h, the gray-level nonuniformity of the COVID-19 pneumonia cases tended to be larger than those of non-COVID pneumonia. There was no significant difference in long-run emphasis, run-length nonuniformity, and short-run emphasis between the two groups (*P* > 0.05).

## Discussion

Chest CT scan can be used for rapid diagnosis of COVID-19 pneumonia. Considering the low specificity of CT scan in the diagnosis of COVID-19 and differentiating it from other types of pneumonia, this study aimed to extract specific radiomics features from non-contrast CT scan images to investigate the ability of textural features in discrimination of COVID-19 pneumonia against other types of pneumonia. In CT radiomics analysis, the mathematical features of lesions are extracted by using spatial parameters and mathematical algorithms to reveal the internal heterogeneity of tissues that cannot be recognized by naked eyes. The texture analysis is the method to evaluate the lesions with gray levels on the image [16, 22].

Recently, there have been efforts to develop the quantitative analysis of CT images for accurate diagnosis of COVID-19. For example, Li et al. developed a fully automatic neural network to detect COVID-19 using chest CT scan and differentiate it from community-acquired pneumonia and other lung diseases. The results of their study showed a high area under the receiver operating characteristic curve (AUC) of 0.95 [23]. In another study by Bai et al., 521 patients with COVID-19 and 665 with no COVID-19 were evaluated. They used the EfficientNet-B4 deep neural network architecture, followed by a two-layer fully connected neural network. The final model of their study achieved an AUC of 0.95 [24]. Wei et al. investigated the ability of textural features to identify the common and severe COVID-19 cases. Twenty significant textural radiomics features were selected in their study which showed good performance in discriminating common and severe COVID-19 cases (AUC > 0.70) [21].

In our study, to reduce the time of radiomics analysis, as a very crucial issue during the COVID-19 pandemic, the ROI was manually localized in a pulmonary consolidation on only one CT image cut. A total of 12 textural features including 8 GLCMs and 4 GLRLMs were extracted to characterize each lesion. The results showed that the cluster tendency, contrast, correlation, dissimilarity, energy, entropy, homogeneity, and gray-level nonuniformity were significantly associated with the COVID-19 status.

Cluster tendency is a measurement of groups of pixels with similar gray-level intensity values. It is clear in Fig. 2a that the values of the cluster tendency in the COVID-19 pneumonia group are much bigger than those in the non-COVID group.

The contrast feature measures the local intensity variations of the image matrix. Table 2 and Fig. 2b indicate that the contrast value of the COVID-19 pneumonia lesions is higher than the other group. This implies a greater difference in intensity values among neighboring pixels in the COVID-19 pneumonia lesions.

As shown in Fig. 2c, the COVID-19 pneumonia lesions have higher correlation values. The linear dependency of gray-level intensities to their respective pixels is measured by the correlation feature. The correlation is a value between 0 and 1. The 0 value indicates uncorrelated pixels and the 1 value indicates the maximum correlation between pixels.

Table 2 indicates that the non-COVID pneumonia lesions were likely to show more homogeneity. This can be clarified that non-COVID pneumonia lesions have more biologically proliferating, and therefore have more pixels with similar uptake that seems to be more homogenous.

Most of the extracted texture features were highly correlated with each other. A homogeneous lesion has higher energy and lower entropy compared to a heterogeneous lesion. The higher the heterogeneity of the lesion, the larger the texture entropy value. Entropy is a measure of the complexity (variability) of the image intensity values. The higher entropy of the COVID-19 pneumonia lesions may indicate that these images are more complex than the non-COVID pneumonia images. The contrast reflects the local intensity variation of pixels that leads to image clarity. So, the high contrast means high clarity of the image. The existence of pairs with different intensity values is measured by the dissimilarity feature. The higher the occurrence of pairs with different values on the image matrix, the higher the dissimilarity and the lower the homogeneity of the lesion. Gray-level nonuniformity evaluates the variability of gray-level intensity values on the image matrix in specific directions. A lower gray-level nonuniformity value indicates a higher homogeneity of the lesion.

Despite the encouraging results, there were some disadvantages in our study. Firstly, this study was retrospective and the sample size was small. It is recommended to use other study designs and a higher number of samples in future studies. Secondly, we used a 2D axial image slice to represent the whole area of the lesion. Texture analysis of 3D isotropic images may lead to better results. Although, the 3D examination of the entire lesion is computationally more complex and time-consuming. Moreover, machine learning-based analyses were not conducted in this study. They can be used in further studies.

## Conclusions

Our study demonstrates the possibility of the use of textural radiomics features in differentiating COVID-19 pneumonia and non-COVID pneumonia lesions in a completely non-invasive approach. Together, the representation of the extracted radiomics features indicated that the COVID-19 pneumonia lesions are more likely to be heterogeneous. Accurate differentiation of COVID-19 pneumonia cases from other types of pneumonia can improve the ability to manage this pandemic.

## Data Availability

Not available.

## Abbreviations

RT-PCR: Real time-polymerase chain reaction
CT: Computed tomography
GGO: Ground-glass opacity
AUC: Area under the receiver operating characteristic curve
ROI: Region of interest
GLCM: Gray-level co-occurrence matrix
GLRLM: Gray-level run-length matrix
